# The Influence of Response Mode on Study Results: Offering Cigarette Smokers a Choice of Postal or Online Completion of a Survey

**DOI:** 10.2196/jmir.1414

**Published:** 2010-10-21

**Authors:** Peter W Callas, Laura J Solomon, John R Hughes, Amy E Livingston

**Affiliations:** ^3^Department of PsychiatryCollege of MedicineUniversity of VermontBurlington, VTUSA; ^2^Office of Health Promotion ResearchUniversity of VermontBurlington, VTUSA; ^1^Medical BiostatisticsUniversity of VermontBurlington, VTUSA

**Keywords:** Internet, mail, questionnaires, smoking

## Abstract

**Background:**

It is unclear whether offering online data collection to study participants affects compliance or produces bias.

**Objective:**

To compare response rates, baseline characteristics, test-retest reliability, and outcomes between cigarette smokers who chose to complete a survey by mail versus those who chose to complete it online.

**Methods:**

We surveyed cigarette smokers who intended to stop smoking within the next 30 days to determine barriers to calling a smoking quit line. Participants were offered the choice of completing a paper version of the survey sent through the mail or an online version at a password-protected website. Participants were called 2 months later to determine if they had made a quit attempt and/or called a smoking quit line since the baseline survey. We compared characteristics and outcomes among those who chose postal versus online completion. We measured test-retest reliability of the baseline survey by resurveying a semirandom sample of participants within 10 days of the original survey.

**Results:**

Of 697 eligible respondents to newspaper ads in 12 US cities, 438 (63%) chose to receive a mailed paper survey and 259 (37%) chose an Internet survey. Survey return rates were the same for the 2 modes (92% versus 92%, *P* = .82). Online respondents were younger (mean of 46 versus 51 years old for postal, *P* < .001), more likely to be white (76% versus 62%, *P* < .001), less likely to be African American (18% versus 30%, *P* < .001), more highly educated (34% college graduate versus 23%, *P* < .001), more likely to intend to stop smoking in the next 30 days (47% definitely versus 30%, *P* < .001), and more likely to have heard of a smoking quit line (51% versus 40%, *P* = .008). Participants did not differ on gender (54% female for online versus 55% for postal, *P* = .72) or cigarettes smoked per day (mean of 19 versus 21, *P* = .30). Online respondents had slightly fewer missing items on the 79-item survey (mean of 1.7% missing versus 2.3%, *P* = .02). Loss to follow-up at 2 months was similar (16% for online and 15% for postal, *P* = .74). There was no significant difference between online and postal respondents in having called a smoking quit line during the 2-month follow-up period (20% versus 24%, *P* = .22) or in having made a quit attempt (76% versus 79%, *P* = .41).

**Conclusions:**

Cigarette smokers who chose to complete a survey using the Internet differed in several ways from those who chose mailed surveys. However, more importantly, online and postal responses produced similar outcomes.

## Introduction

Since the origin of the World Wide Web, its potential for use in research studies has been recognized. One use is to collect information from study participants [1]. This method can be less expensive and produce data sooner with fewer errors. However, due to nonuniversal Internet access and dissimilarity in the physical nature of how data are collected from the World Wide Web as compared to more traditional methods, there are concerns about potential systematic differences in data collected by these methods [2,3].

A number of randomized and nonrandomized studies have compared postal and online responses regarding alcohol and drug use, particularly among college students [4-9]. Few such studies have been conducted among cigarette smokers in particular [10,11], with none having examined the effect of giving these respondents a choice of survey mode.

We conducted a prospective study among cigarette smokers who intended to quit smoking to identify barriers to calling a toll-free quit smoking phone line [12]. In this study, participants chose to complete a survey using either a paper questionnaire returned by mail or an online survey accessed at a secure website. The primary aim of the current analysis was to determine if study outcomes differed for cigarette smokers who chose different data collection methods. If they did differ, this could be an indication of potential selection or information bias. Secondary outcomes were to compare participant characteristics and test-retest reliability of those who chose paper or online questionnaires.

## Methods

### Sample

Potential participants were recruited in 2007 using newspaper advertisements in 12 US cities in 8 states. The advertisement was as follows:

Daily cigarette smokers who plan to quit smoking wanted for University of Vermont research study. This study does not offer treatment. Compensation for completing mailed or online survey about quit smoking services and one brief follow-up phone call. If interested, call 1-800-[xxx-xxxx] (toll-free).

Screening for eligibility was obtained over the phone. Eligible participants were at least 18 years of age, fluent in English, smoked at least 5 cigarettes daily, intended to quit in the next 30 days, and had not called a smoking quit line in the past 30 days. Verbal informed consent was obtained from all participants during the screening phone call after the participant had been determined to be eligible. The consent statement included that the study was funded by the National Institutes of Health, and stated that the study:

…involves filling out a 20-minute mailed or online survey about your cigarette smoking and your thoughts about services that might help you quit, and then completing a 5-minute interview by telephone about two months later. We will reimburse you [US] $35 for the survey and [US] $25 for the telephone interview. You may or may not be asked to fill out the 20-minute survey a second time. If you are asked to do that, you will be reimbursed an additional [US] $35.

The statement also indicated that all information would be confidential, participation was voluntary, and the participant could refuse at any time. Participants were provided with the name, phone number, and email address of the principal investigator (author JRH). Confidential data were stored on a password-protected computer with access limited to study personnel. The study was approved by the University of Vermont Institutional Review Board. All study personnel were required to complete a tutorial from the University of Vermont Institutional Review Board on the protection of human subjects in research.

### Instruments

At the conclusion of the initial phone call, participants were given a choice of completing a baseline survey via returning a mailed paper version in a prepaid envelope or accessing an online version using a password protected website. Participants were asked, “Would you prefer that we mailed you the survey through regular mail with a stamped return envelope or would you rather complete the survey online?”

The baseline survey asked demographic and smoking information and an additional 53 items specifically targeting barriers to calling a smoking quit line (eg, “I might not call the quit line because I’m sure I can quit on my own,” with response choices: 1 = not at all true for me; 2 = somewhat true for me; 3 = mostly true for me; 4 = completely true for me). The only previously validated items on the survey were a subset of questions from the Fagerstrom Test for Nicotine Dependence [13]. The survey contained a total of 79 items, formatted with contrasting shading for every other item so that respondents could distinguish them easily. The printed version was 6 pages long.

The Web version of the questionnaire was formatted to look the same as the paper version. To avoid rapid online responses, there were no more than 5 items per screen, giving a total of 16 screens. For consistency with the paper survey, online respondents could skip items and could go back to review responses before submitting the survey. All submitted questionnaires were included in the analysis even if items were missing, but questionnaires terminated before submission were not included. Multiple submissions by the same subject were not allowed—each respondent who chose to complete the survey online received a password that could only be used for one submission.

The online system was tested in 2 pilot studies that were conducted to construct the 53 items concerning barriers to calling a smoking quit line, and the final online questionnaire was completed multiple times by members of the research team before any participants were enrolled.

Because of concern of respondent fatigue, 10 versions of the baseline survey were used, with the 53 items concerning barriers in a different order for each. No significant differences in the means or variances of the items were found, so the 10 versions were pooled for the analysis.

A follow-up telephone survey 2 months later asked whether participants had called a quit line or had made a quit attempt since completing the baseline survey. Test-retest reliability of the baseline survey was assessed by requesting a semirandom sample of the postal and online respondents to repeat the survey 10 days after original completion.

### Statistical Analysis

Postal and online respondents were compared using chi-square tests for categorical variables and the Wilcoxon rank sum test for continuous variables. A nonparametric approach was used for comparing continuous variables due to the nonnormal distribution of several of the variables. Test-retest reliability was measured by computing intraclass correlation coefficients for consistency using a two-way random-effects model [14], which measures repeatability of responses. Differences in 2-month results (incidence of calling a quit line or making a quit attempt) were compared using chi-square tests for bivariate analyses and logistic regression to control for baseline differences in respondents. Analyses were conducted using SAS 9.1 (SAS Institute Inc, Cary, NC, USA) except for reliability, for which SPSS 15.0 (SPSS Inc, Chicago, IL, USA) was used.

## Results

Results pertaining to barriers to calling a smoking quit line have previously been reported [12]. Overall, 1527 people called in response to the newspaper advertisements and were screened. Of these, 789 (52%) were ineligible, with most (691/789, 88%) excluded because they did not answer “probably” or “definitely” to the question “Are you planning to quit smoking cigarettes in the next 30 days?” An additional 41 callers did not give verbal consent, leaving 697 recipients of the baseline survey.

**Figure 1:**
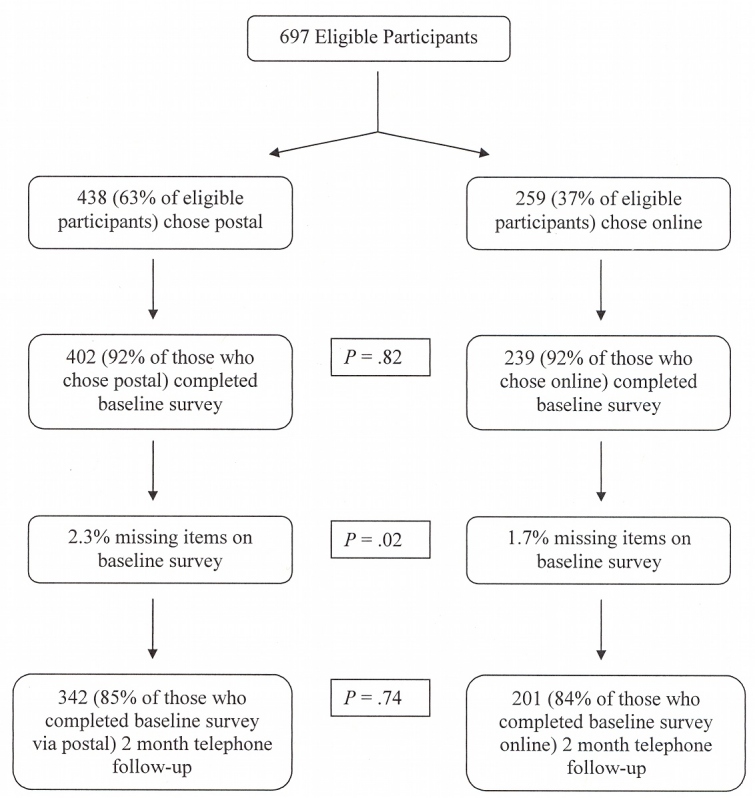
Choice of response mode, response rates, and rates of missing items

Of the 697 participants, 438 (63%) chose to complete the baseline survey using mail (Figure 1). Surveys were returned by an equal percentage of those who chose mail and those who chose Internet (χ^2^_1_ = 0.1, *P* = .82). There were significantly more missing items on the paper survey but the difference was very small (χ^2^_1_ = 5.7, *P* = .02). Of those who completed the baseline survey, a similar proportion of postal and online respondents completed the 2-month follow-up phone survey (χ^2^_1_ = 0.1, *P* = .74).

Comparisons of baseline characteristics and responses are shown in Table 1. There was no difference in gender for postal versus online completers. Online respondents were significantly younger and more educated. African Americans were more likely to use a paper form, while a higher percentage of whites chose online. (There are separate *P* values for each ethnic group because respondents could choose more than one ethnic group.) The number of cigarettes smoked per day was similar. Online respondents were more likely to definitely intend to stop smoking in the next 30 days and were more likely to have heard of a smoking quit line but were equally likely to have called a quit line in the past. Importantly, the mean scores on the 53 barriers items (the major independent variable) were the same.

**Table 1:** Comparison of postal and online responses on baseline survey

				Test Statistic (χ2)		
		Postal	Online	df	χ2	*P* value
Total n		402	239			
Age in years, mean (SD)		51 (12)	46 (13)	1	28.5	< .001
Female gender, n (%)		222 (55%)	128 (54%)	1	0.1	.72
**Highest level of education**						
	≤ High school, n (%)	154 (38%)	43 (18%)	2	30.0	< .001
	Some college, n (%)	153 (38%)	114 (48%)			
	College degree, n (%)	94 (23%)	82 (34%)			
**Ethnicity**						
	Hispanic, n (%)	33 (8%)	15 (6%)	1	1.0	.32
	African American, n (%)	120 (30%)	43 (18%)	1	11.2	< .001
	White, n (%)	249 (62%)	181 (76%)	1	12.6	< .001
	Other, n (%)	45 (11%)	18 (8%)	1	2.3	.13
Cigarettes per day, mean (SD)		21 (12)	19 (10)	1	1.1	.30
**Do you intend to stop smoking in the next 30 days?**						
	Definitely not, n (%)	0 (0%)	0 (0%)	3	21.8	< .001
	Probably not, n (%)	22 (6%)	7 (3%)			
	Possibly, n (%)	149 (37%)	59 (25%)			
	Probably, n (%)	110 (28%)	61 (26%)			
	Definitely, n (%)	119 (30%)	112 (47%)			
**How confident are you that you can stop in next 30 days?**						
	Not at all confident, n (%)	56 (14%)	26 (11%)	4	9.6	.05
	Slightly confident, n (%)	184 (46%)	91 (38%)			
	Confident, n (%)	101 (25%)	70 (29%)			
	Very confident, n (%)	46 (11%)	32 (13%)			
	Extremely confident, n (%)	15 (4%)	19 (8%)			
Have you ever heard of a smoking quit line? Yes, n (%)		162 (40%)	122 (51%)	1	7.0	.008
Have you ever called a smoking quit line? Yes, n (%)		35 (9%)	16 (7%)	1	0.8	.36
Mean of 53 barriers items, each on scale of 1 to 4 (1=not at all true for me, 4=completely true for me), mean (SD)		1.6 (0.5)	1.6 (0.4)	1	0.03	.87

**Table 2:** Comparison of postal and online baseline respondents at 2-month telephone survey

			Test Statistic (χ2)		
	Postal	Online	df	χ2	*P* value
Total n	342	201			
Called smoking quit line, n (%)	82 (24%)	39 (20%)	1	1.5	.22
Made quit attempt, n (%)	270 (79%)	153 (76%)	1	0.7	.41

To measure test-retest reliability, the baseline survey was repeated by 55 (74%) of 74 postal and 27 (63%) of 43 online participants invited to retake the survey. The intraclass correlation coefficient was .76 (95% confidence interval [CI] .61-.85) for postal and .90 (95% CI .80-.95) for online.

At 2-month follow-up, slightly more postal respondents had called a smoking quit line and had made a quit attempt, but these differences were not statistically significant (Table 2). Since true differences could be masked by confounding due to baseline differences, these comparisons were repeated using logistic regression to adjust for age, education, and ethnicity. The adjusted results were essentially the same as the bivariate findings shown in Table 2.

## Discussion

Our major finding is that although online participants varied in some ways from postal participants, these differences did not appear to affect the study results. Response rates, missing data, reliability, and follow-up rates were at least as good for online participants as for postal participants, and outcomes at 2 months were similar for the 2 groups.

Study participants who chose to complete the baseline survey online were, on average, younger, better educated, less likely to be African American, and more likely to be white. Given the demographics of Internet use [15], these differences are not surprising. We also found those who chose Internet were more likely to intend to quit, although actual quitting was not greater in this group. An analysis of a nationally representative sample found similar results for age, education, ethnicity, and gender for smokers who do and do not use the Internet and also found that smokers who use the Internet were more likely to report planning to quit smoking [16]. In a comparison of postal, Internet, and telephone respondents to the Behavioral Risk Factor Surveillance System, which asks about smoking and other risk behaviors, there were no differences in gender and ethnic distribution for Internet respondents [4]. A survey of alcohol use among college students found no ethnic differences in online and postal respondents, but found online respondents to be younger and more likely to be male [7]. However, these differences may be because the option of completing the survey via mail was only offered to nonrespondents of the Internet invitation. Another study that used this design found online respondents to have higher mean education and income than postal respondents [17], which could be due to better computer access for those with higher education and income or could be because of higher education of first responders in general.

We did not find a difference in response rates between the online and Internet groups. Response rates might be expected to be higher for online participants due to the more immediate receipt of the survey, but could be expected to be lower because of the lack of a physical reminder of the survey (eg, paper survey sitting on the kitchen counter) and because the email with the password and website could be deleted as potential spam. Past studies have had mixed results, with some having lower Internet response rates [18-22], some with no difference [9,23,24], and some with higher Internet response rates [5,6,25]. These studies differed from ours in that participants were randomized to condition rather than given a choice, which would affect comparability of response rates.

Meta-analyses comparing Web and postal response rates find overall lower response rates for Web surveys [26,27], but at least some of the studies included in these meta-analyses recruited subjects at websites or through the mail, rather than recruiting all subjects using the same method as in our study.

In our study, the high response rates for both groups may be due to (1) participants who were motivated enough to call in response to a newspaper advertisement and/or (2) financial incentives for completing the survey. Both monetary and nonmonetary incentives have been found to substantially increase response rates [28].

Test-retest reliability of the survey was at least as good for online participants and perhaps better. If it was truly better, this could be due to the differing characteristics of the respondents (eg, education). Other studies have found high reliability for Internet questionnaires completed by smokers, with no apparent systematic differences from other modes [10,11] and other populations [8,19,23,29-38]. Im et al [39] found higher reliability and convergent validity for postal as compared with online completion of the Midlife Women’s Symptom Index; however, they concluded that reliability and validity were sufficiently high for both formats.

Responses on the survey may have been influenced by social desirability bias, where participants may have overreported factors such as desire to quit smoking. Whether such bias differentially affects online versus postal responses is unclear. Our finding of higher baseline intentions to stop smoking and confidence in ability to stop in online respondents could be due to higher social desirability bias in this group or could be due to demographic differences between the groups. Randomized studies of alcohol use [8] and illicit drug use [5] did not find differences in reporting of these behaviors for different modes of survey completion.

Our finding of low rates of missing data for both survey modes agrees with the results of Smith et al [40], who found a mean number of missing items of 1.7% for both online and postal respondents. Their study was similar to ours, with participants choosing to return the survey online or through the mail. In a survey of college students on alcohol and other drug use [6], surveys returned via Internet and mail both had 2.6% missing data rates. Surveying pediatricians, McMahon et al [41] found significantly fewer missing items for surveys returned via email as compared with mail or fax, but the mean number of missing items was low for all three modes (0.4%, 2.1%, and 2.8%, respectively). Im et al [39] observed similar findings surveying women aged 40 to 60 years, with 1.3% of items missing for Internet completion and 2.6% missing for mailed surveys.

A limitation of our study is that participants were not randomized, but self-selected survey mode. Many prior studies have randomized respondents to complete surveys by paper or online [5,6,8,18,19,21-25,29,38,42] or have used a within-subject design where each participant filled out both paper and online forms [10,11,29-32,37]. These approaches have the advantage that any observed differences are likely due to mode of completion. Although a randomized design would have been possible for this study, our intent was to compare the characteristics of those who chose to use Internet with those who chose mail, since this is the design used for many studies that include an Internet option. Our observational design allowed examination of the actual circumstances under which such Internet surveys are implemented, which increases external validity.

Our external validity may be decreased, however, by using a volunteer sample recruited from newspaper advertisements. Compared with all current daily smokers in the United States [43], our respondents were somewhat older (mean of 49 years versus 42 years), more likely to be female (55% versus 46%), better educated (69% with education beyond high school versus 39%), more likely to be African American (25% versus 11%), and smoked more (mean of 20 cigarettes per day versus 16). Some of these differences may be due to recruitment methods, and some may be due to the eligibility requirements of the study (eg, smoke at least 5 cigarettes per day and intend to quit in the next 30 days).

Offering participants a choice of postal or online completion of a survey can gain some of the advantages of Internet use in research while avoiding some of the disadvantages. Internet surveys are generally less expensive than mail, have faster response times, and have the potential for more valid data by automated skip patterns and checks for illogical values [2,3]. However, recruiting participants via the Internet can suffer from low response rates and questions about who is missed due to lack of Internet access [2]. These are not issues when participants are recruited using traditional methods but given a choice of response mode.

A number of studies have reported consistency in research results across online and postal response formats in spite of differences in respondent characteristics [3]. We have extended this finding to a sample of cigarette smokers. In summary, we found that offering online and mail versions of a survey allowed participants to choose whichever was most convenient without having a negative impact on the study data.
